# Referrals to a critical care unit: compliance with the NCEPOD recommendations

**DOI:** 10.1186/cc13197

**Published:** 2014-03-17

**Authors:** G Wigmore, M Campbell, P Walker, K Raveendran

**Affiliations:** 1 The Princess Alexandra Hospital NHS Trust, Harlow, UK; 2 Barking Havering and Redbridge University Hospital, London, UK

## Introduction

The report of the National Confidential Enquiry into Patient Outcome and Death (NCEPOD) 2005 [1] provides recommendations to reduce morbidity and mortality among acutely ill patients. It highlights the importance for involvement of consultants in the referral process, clear physiological monitoring plans for each patient, and review by a consultant intensivist within 12 hours of admission to critical care. The objective of the audit was to assess compliance with these recommendations, ensuring safe management of acutely unwell patients and appropriatetilisation of scarce critical care resources.

## Methods

Prospective data on referrals to adult critical care in a 20- bed unit in a teaching hospital were collected over 8 weeks. Collected data included: source, time, seniority of doctor referring and receiving referral, outcome of referral, involvement of team consultant prior to referral, documented management plan and review by a critical care consultant within 12 hours of referral.

## Results

Seventy-three referrals were analysed, the majority of which were medical. One-half of referrals came out of hours; 24% of referrals were made by a consultant; 51% were seen by a consultant prior to referral; 73% of referrals were admitted; likelihood of admission increased from 63% to 83% if the patient was reviewed by home consultant prior to referral. In 56% of cases the referral was received by a foundation doctor (all referrals were discussed with a consultant intensivist). Among the rejected referrals (maximal ward therapy not reached), 54% were from a trainee below registrar grade. Twenty-five per cent of patients were not seen by a consultant intensivist within 12 hours of referral.

## Conclusion

Acutely unwell patients require the expertise of the most senior clinicians regarding further management, including planning for end-of-life care. Our audit demonstrated poor adherence to NCEPOD [1] and Department of Health [2] recommendations. The majority of referrals to critical care were made by nonconsultants and for patients who had not been reviewed by a team consultant, prior to referral. The workload in critical care demonstrated that almost one- half of the referrals happen out of hours. These findings have resulted in significant changes to working practice, including the presence of an onsite consultant intensivist for a minimum of 13 hours daily.
